# Supplementary material for: Human brain region-specific variably methylated regions are enriched for heritability of distinct neuropsychiatric traits
Source: Genome Biol. 2021 Apr 22;22:116. doi: 10.1186/s13059-021-02335-w (PMC8061076; doi:10.1186/s13059-021-02335-w)
Supplement: Supplementary file 1 — Additional file 1. Supplementary Materials including Supplementary Figures and Description of Supplementary Tables. [file 13059_2021_2335_MOESM1_ESM.pdf]

Additional Materials

Supplementary Figures

1. Figure S1 Isolation and characterization of neuronal (NeuN+) nuclei from frozen brain tissue samples.
2. Figure S2 Validation of neuronal proportions and variability measurements.
3. Figure S3 ChromHMM enrichment and distribution of VMRs.
4. Figure S4 Distribution of ubiquitous VMRs across the genome.
5. Figure S5 Neuronal heterogeneity among subregions of the amygdala contribute to methylation variability.
6. Figure S6 SLDSR analysis of DMRs and VMRs.

Supplementary Tables

1. Table S1 Sample phenotype data.
2. Table S2 WGBS sequencing statistics.
3. Table S3 Samples failing genotype quality control check.
4. Table S4 CG-DMRs identified among neuronal samples isolated from all 8 brain regions.
5. Table S5 CG-DMRs identified among the 5 brain region groups (cortical, basal ganglia, hypothalamus, hippocampus, amygdala).
6. Table S6 Large blocks of differential CpG methylation identified among the 5 brain region groups (cortical, basal ganglia, hypothalamus, hippocampus, amygdala).
7. Table S7 CG-DMRs identified among neuronal samples isolated from the basal ganglia tissues (caudate, putamen, nucleus accumbens).
8. Table S8 GREAT analysis of basal ganglia CG-DMRs unique to this analysis and the top 2,000 Hippocampal CG-DMRs.
9. Table S9 CG-DMRs identified among neuronal samples isolated from the two hippocampus tissue groups.
10. Table S10a List of 75 granule cell marker genes (hg38 coordinates).
    Table S10b Hippocampal CG-DMRs that overlap granule cell marker genes or their promoters (TSS +/- 4 kb).
11. Table S11 CH-DMRs identified among the 5 brain region groups (cortical, basal ganglia, hypothalamus, hippocampus, amygdala).
12. Table S12a CH-DMRs identified among neuronal samples isolated from the basal ganglia tissues.
    Table S12b CH-DMRs identified among neuronal samples isolated from the two hippocampus tissue groups.
13. Table S13 Lists of VMRs identified in each tissue.
    - All_VMRs: Union of all VMRs from all tissues (9 brain regions, lung, and thyroid).
    - Ubiquitious VMRs: VMRs shared across all tissues (9 brain regions, lung, and thyroid).
    - Dentate gyrus all: All VMRs identified in the hippocampus (dentate gyrus) samples.
    - Dentate gyrus only: Tissue-specific VMRs identified only in the hippocampus (dentate gyrus) samples.
    - HC only: Tissue-specific VMRs identified only in the hippocampus samples.
    - HC all: All VMRs identified in the hippocampus samples.
    - AMY only: Tissue-specific VMRs identified only in the amygdala samples.
    - AMY all: All VMRs identified in the amygdala samples.
    - HYP only: Tissue-specific VMRs identified only in the hypothalamus samples.
    - HYP all: All VMRs identified in the hypothalamus samples.
    - NAcc only: Tissue-specific VMRs identified only in the nucleus accumbens samples.
    - NAcc all: All VMRs identified in the nucleus accumbens samples.
    - PUT only: Tissue-specific VMRs identified only in the putamen samples.
    - PUT all: All VMRs identified in the putamen samples.

BA9 only:	Tissue-specific VMRs identified in the frontal cortex (BA9) samples.
BA9 all:	All VMRs identified in the frontal cortex (BA9) samples.
BA24 only:	Tissue-specific VMRs identified in the frontal cortex (BA24) samples.
BA24 all:	All VMRs identified in the frontal cortex (BA24) samples.
CAU all:	All VMRs identified in the caudate samples.
CAU only:	Tissue-specific VMRs identified only in the caudate samples.
Thyroid only:	Tissue-specific VMRs identified only in the thyroid samples.
Thyroid all:	All VMRs identified in the thyroid samples.
Lung only:	Tissue-specific VMRs identified only in the lung samples.
Lung all:	All VMRs identified in the lung samples.

14. Table S14 GREAT analysis of VMRs shared only between amygdala and hypothalamus samples.
15. Table S15 Links and references to summary statistics for 30 traits used in stratified linkage disequilibrium score regression analyses.
16. Table S16 Results from stratified linkage disequilibrium score regression analyses using epigenetic features.
17. Table S17 Overlap between VMRs and SNPs at several minor allele frequencies.

FIGURE S1

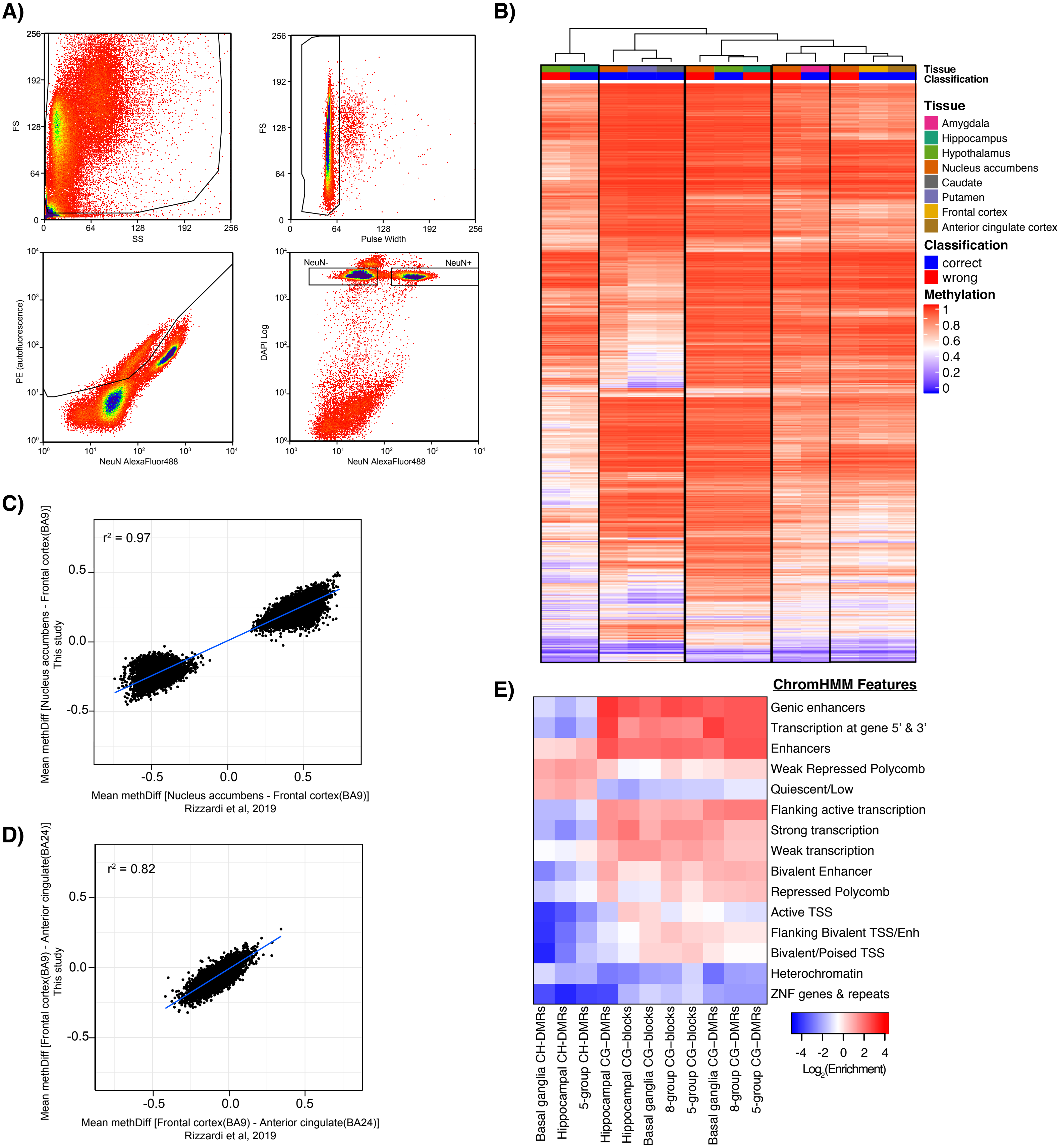

Figure S1. Isolation and characterization of neuronal (NeuN+) nuclei from frozen brain tissue samples. (A) Nuclei in this representative example from caudate tissue were isolated by fluorescence activated nuclei sorting from caudate and debris, doublets, and auto-fluorescent nuclei were gated out as shown. The remaining nuclei were separated based on detection of AlexaFluor 488-conjugated anti-NeuN antibody (MAB377X). (B) Several samples were outliers based on principal component analysis and were eliminated from further analysis. To confirm that they were incorrectly labeled, we averaged the methylation values from all other samples in each tissue group over the CG-DMRs identified among the 5 tissue groups. We then performed hierarchical clustering of the outlier samples (red) along with the merged tissue groups (blue). This analysis revealed the most likely tissue of origin for these outlier samples and confirmed their incorrect labeling. C) Comparison of methylation differences at DMRs identified in [12] between nucleus accumbens and frontal cortex (BA9) NeuN+ samples from [12] and this study. D) Comparison of methylation differences between frontal cortex (BA9) and anterior cingulate cortex (BA24) NeuN+ samples from [12] (x-axis) and this study (y-axis) at all 13,074 DMRs identified in [12]. E) Heatmap representing mean log2 enrichment of all CG-DMRs, CG-blocks, and CH-DMRs identified among the 5 tissue groups as indicated compared to the rest of the genome for chromHMM states from 4 brain regions (15-state model).

FIGURE S2

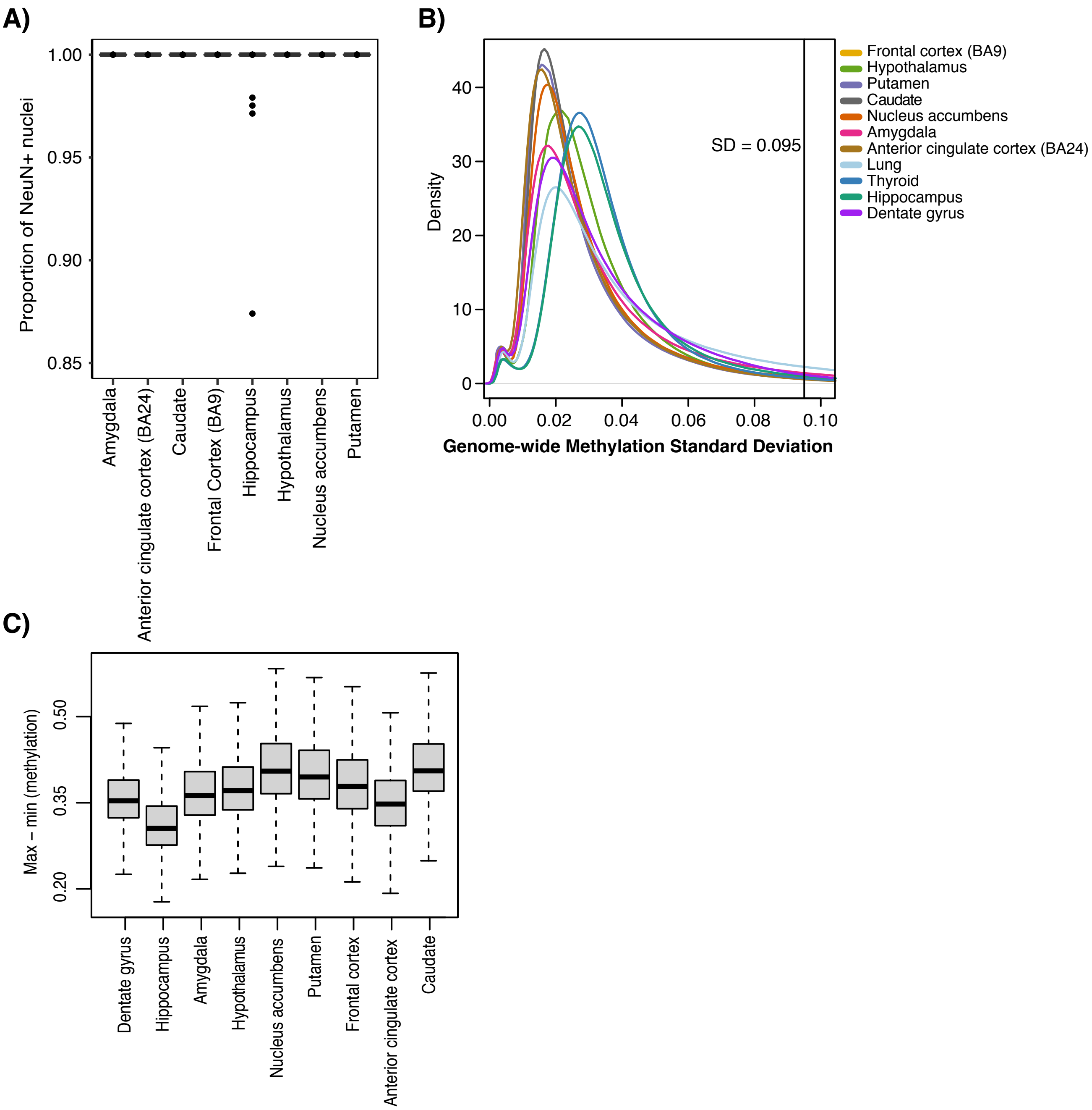

Figure S2. Validation of neuronal proportions and variability measurements. A) Estimates of neuronal proportions using EpiDISH. B) Distribution of standard deviation values within each tissue with the VMR cutoff value indicated. C) The distribution of VMR effect sizes, defined as maximum – minimum of the per-region, sample specific methylation.

FIGURE S3

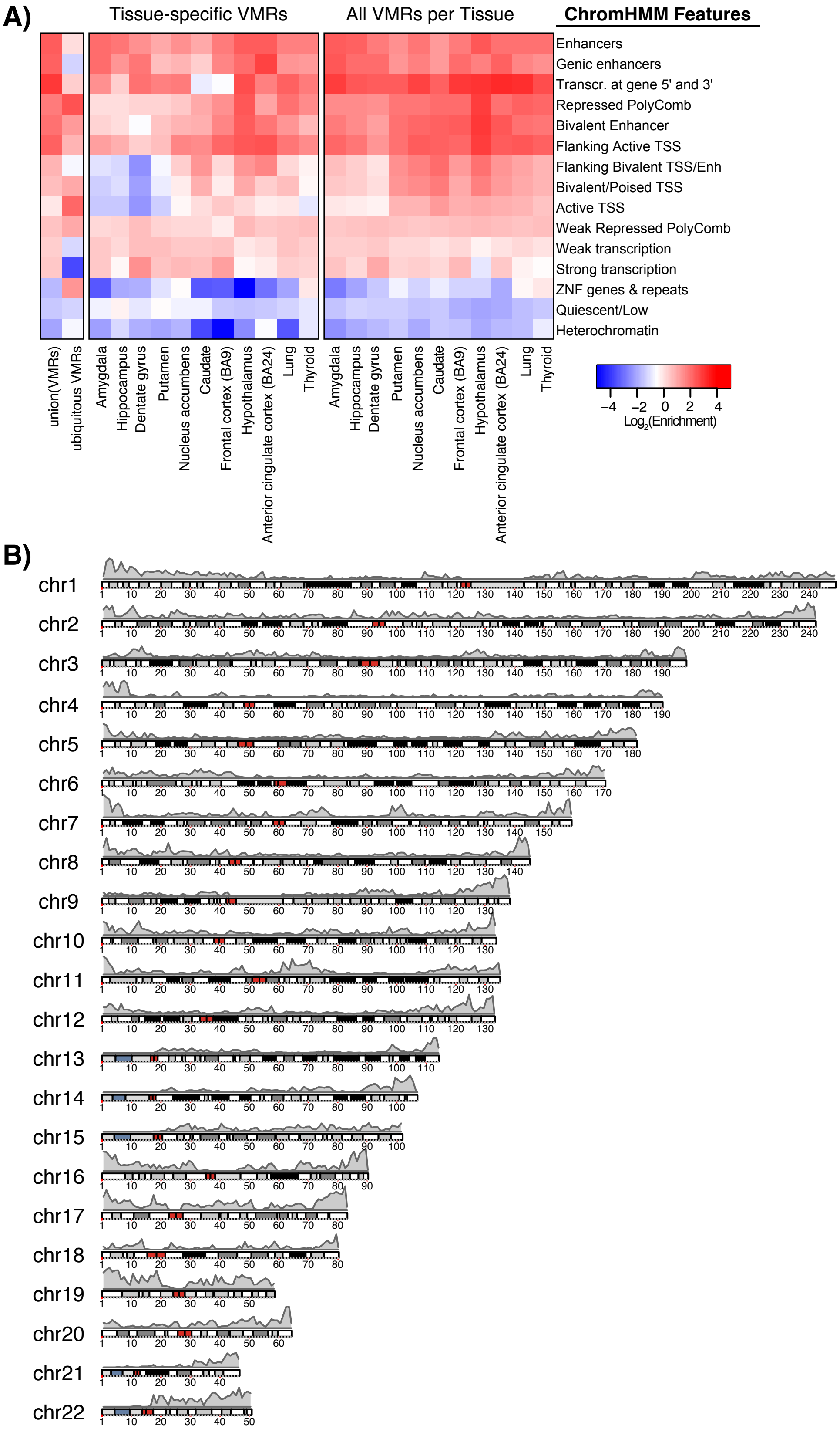

Figure S3. ChromHMM enrichment and distribution of VMRs. A) Heatmap representing log2 enrichment of the union of all VMRs identified. ubiquitous VMRs, tissue-specific VMRs, and all VMRs identified in each tissue as indicated compared to the rest of the genome for chromHMM states from 4 brain regions (15-state model). B) Distribution of all VMRs across the genome.

FIGURE S4

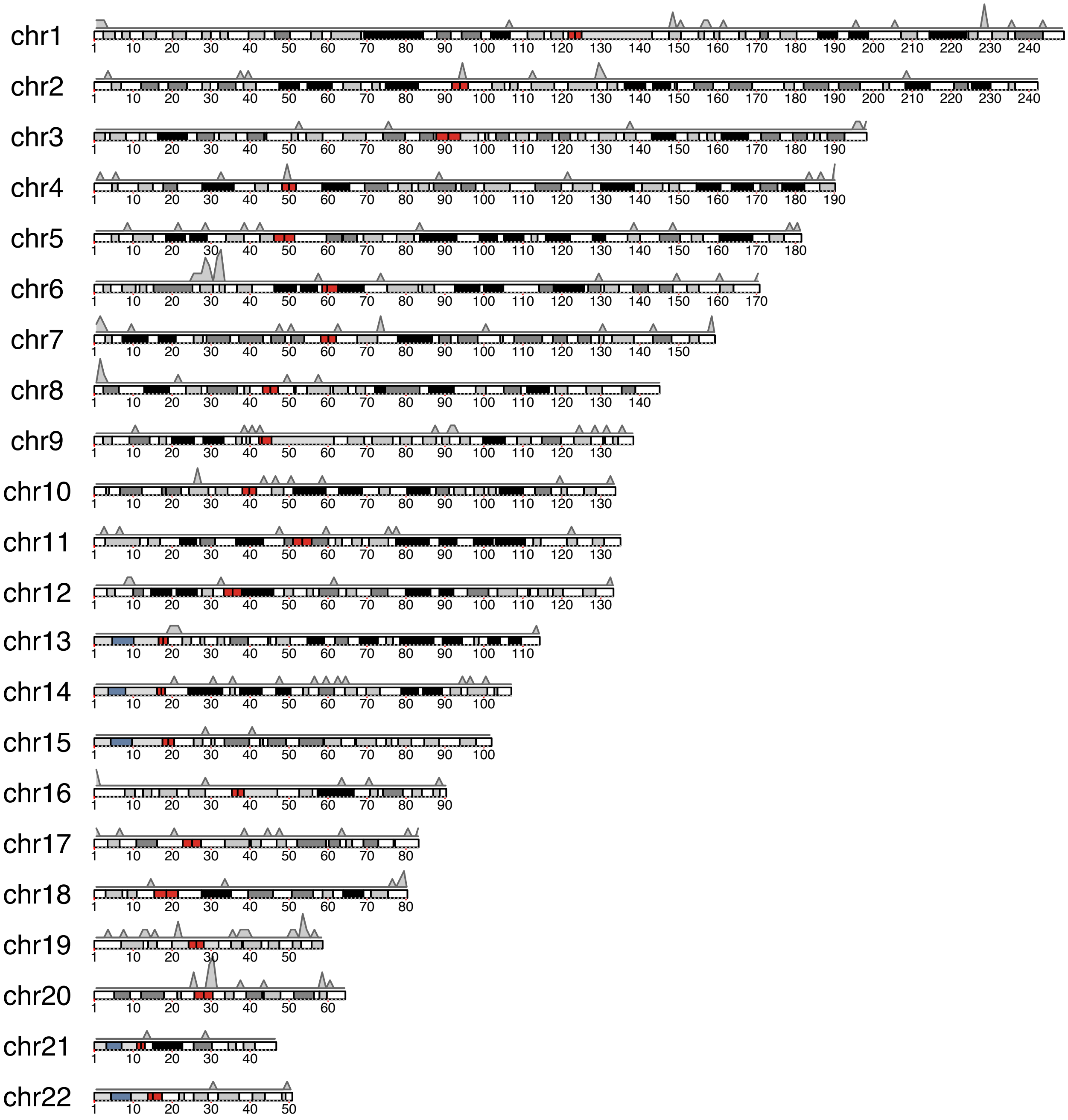

Figure S4. Distribution of ubiquitous VMRs across the genome.

FIGURE S5

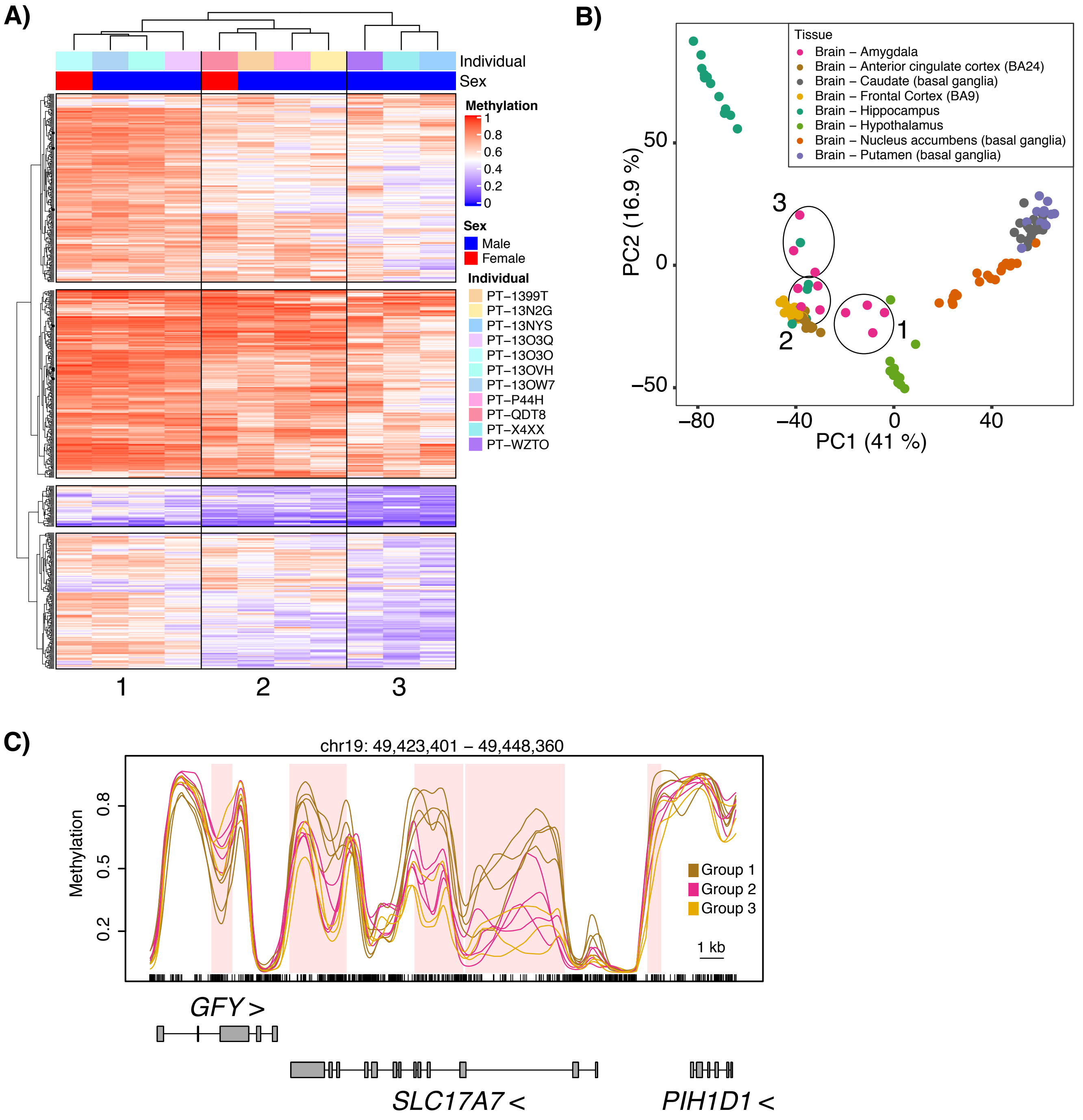

Figure S5. Neuronal heterogeneity among subregions of the amygdala contribute to methylation variability. (A) Hierarchical clustering of amygdala samples based on the average methylation in the 649 VMRs identified in amygdala within 1 kb of the human homologs of genes that identify distinct subregions within the amygdala. (B) Identification of the three amygdala subgroups on the initial principal component analysis from Figure 1A. (C) Example VMRs within the SLC17A7 gene showing average methylation values for each amygdala subgroup identified in B as indicated. VMRs are shaded pink.

FIGURE S6

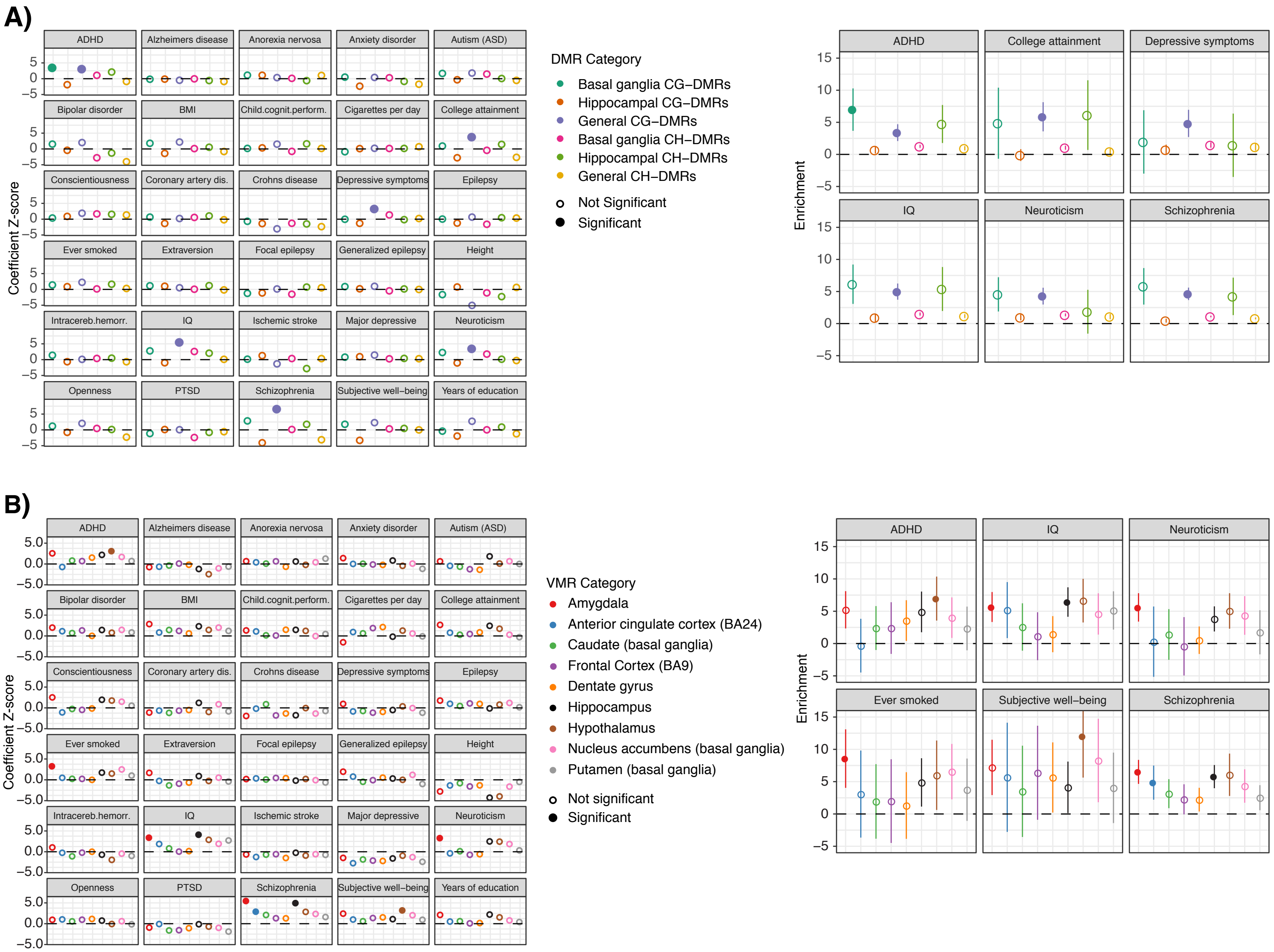

Figure S6. DMRs and VMRs enriched for heritability of neurological traits. Results from running stratified linkage disequilibrium score regression using 30 GWAS traits with the (A) DMRs and (B) VMRs identified in this study and 97 baseline features. Z-scores for each trait are shown (left) and enrichments +/- 2 standard errors are shown (right) only for traits with at least one significant association.
