# Supplementary material for: Human brain region-specific variably methylated regions are enriched for heritability of distinct neuropsychiatric traits
Source: Genome Biol. 2021 Apr 22;22:116. doi: 10.1186/s13059-021-02335-w (PMC8061076; doi:10.1186/s13059-021-02335-w)
Supplement: Supplementary file 19 — Additional file 19. Review history. [file 13059_2021_2335_MOESM19_ESM.docx]

Review History

**First round of review**

**Reviewer 1**

**Are you able to assess all statistics in the manuscript, including the appropriateness of statistical tests used?** Yes, and I have assessed the statistics in my report.

**Comments to author:**

Manuscript Number: GBIO-D-20-01078

Title: Human brain region-specific variably methylated regions (VMRs) are enriched for heritability of distinct neuropsychiatric traits

I think this paper is worthy of publication. However, my main issue with the current format is that I find much of the way the data is presented rather impenetrable, and it took me multiple reads of the paper to digest it all and figure out some of the things that were being presented. I found that several of the figures are displayed in ways that make it very hard for the reader to figure out what they are actually showing. Acronyms are used very frequently, and are often completely lacking from the figure legends. At points I sometimes felt I had to learn a new language to try and understand what was being displayed when looking at the some of the figures with all the acronyms that were used... I realize what the authors have done here encompasses multiple tissues, both CpG and non-CpG methylation, and analysis of both differential (fixed) and variable (polymorphic) methylation, but in its current form it is at points so thick with acronyms that you have to think really hard about some sentences and figures to decipher what is being stated or shown. I therefore think that the authors need to work on ways of making these data a bit more accessible to the reader. I know there is a balance to be made of using acronyms to save repetition and word count, but I would like to see a revision in which they try to ensure figures can be understood as stand-alone objects without being forced to comb through the text (I think this is important as readers often look at figures without reading everything), and perhaps removing some of the acronyms used, particularly for items that are not stated very often.

Specific Comments

While the methodology used to define DMRs is explained in the Methods section, I think it would be of benefit to briefly state what was done at the beginning of the Results when DMRs are first mentioned. This would give the reader some context of what these are before then talking about how many DMRs were found.

Line 127, Results: Authors state "CG-DMRs encompassed 99.5% 128 (13019/13074) of the CG-DMRs we previously identified [11]." This statement seems either unnecessary, or needs a bit more explanation. To make it clearer, if the authors here are trying to indicate that there is very strong agreement between the results in this present study and a previous one, I think it would be best to state exactly that. Otherwise, as written, this statement is rather hanging and unconnected to anything else before or after.

In the abstract, the authors state they identified conventional differentially methylated regions (DMRs)". The term DMR is used in many different contexts in the literature, e.g. to notate regions that have differential methylation between samples or cohorts (e.g. case vs control studies), in relation to imprinted loci (i.e. differential methylated between parental alleles), or what I suspect the authors mean here, which is differential methylation among tissues. I think they should state clearly what the "differential" of "differential methylation" is referring to when used in this manuscript.

Similarly in the introduction, it is stated "enabled our identification of variably methylated regions (VMRs)". Again, this term is used without definition, which I think is key to enable most readers to understand clearly what the study is doing. Here VMR is used by the authors to refer to regions of variable methylation observed within a single tissue among different individuals (ie inter-individual polymorphism), but this needs to be explicitly defined when first talked about.

Results: The authors state "we generated >30B uniquely mapped 150bp paired end reads". Instead of B, better to say billion?

Table 2 displays the fraction of VMRs that overlap SNPs of varying allele frequencies. This seems not a very useful result to me, as this is a very crude measure, and it's not quite clear what the point of this table is. My guess is the authors are trying to show that ubiquitous VMRs have a slightly increased fraction of common SNPs, but this is never commented on, and not formalized in any way (is it significant?). Even if this is the point and it is significant, I think it would be better simply stated in the text, and does not require a dedicated table in the manuscript (maybe Supplementary would be better?). To hammer this home, the authors then go on to identify SNPs associated with VMRs, and look +/-50kb around (not just overlapping), indeed finding many associated SNPs which lie outside the VMRs. Thus, the relevance of SNPs directly overlapping VMRs is diminished.

I find Figures 1c and 1d rather confusing (especially 1d). CG-blocks are not explained/defined, and there is only one very brief mention of them in the text of the Results, so it is not clear to me what these are exactly (I'm guessing large chunks of differential methylation?). I think part of the issue is the notation. What is HC, BG and All? I eventually found them listed in the legend, but why not just write hippocampal and basal ganglia in the plot? The color scale could be much better labeled, as "log2OR" is not clear. Why not say "log2 enrichment" (I presume OR=Odds Ratio?)? But in 1d I am also confused as to how there can be HC (I think this means Hippocampal) on the x axis, but then the y-axis also includes Hippocampus?

Fig1d shows "OCRs', but these are not explained. I managed to track this down to be "Open Chromatin Regions", but OCR is never defined anywhere in the entire manuscript as far as I could see

Fig2A. The format used to show the color/study is confusing. Why not just use 3 different symbols rather than a combination of color and shape, which I found hard to disentangle. Also surely there is a better notation than "NatNeuro", which is very generic and ambiguous. eg. "Rizzardi et al 2019"?

Figure 5: This figure shows "BG CG DMRs" and "CG DMRs". Although they do define in the text, neither are defined (or even mentioned) in the figure legend. It would be better to not use an acronym for BC here. If someone simply looks at the figures, it is impossible to understand what is being shown without first digging through the text to track down where these are defined.

Figure 6 has way too many acronyms, none of which are explained to the reader. The x-axis of Fig 6c has reduced different cell types (I think?) to 2 or 3 letter codes, but these are not at all clear. Furthermore there are other things listed such as nVMRs - what is an nVMR, and how is it different to a VMR? What is a ubiquitous VMR? I don't see what the left heat map panel adds, as it should be obvious that nVMRs overlap a lot with All VMRs or ubiquitous VMRs. Then I am also not clear what is the difference between the other two panels, which are labeled "Tissue Specific VMRs" and "All VMRs per tissue"? I not sure I understand what is being presented here.

**Reviewer 2**

**Are you able to assess all statistics in the manuscript, including the appropriateness of statistical tests used?** Yes, and I have assessed the statistics in my report.

**Comments to author:**

This study uses samples from GTEx to assess differences in DNA methylation between brain regions and individuals. Although tackling an important question, overall the analyses presented in this manuscript are not particularly novel or ground-breaking. It is not surprising that DNA methylation varies across different regions (and cell-types) of the human brain - this has been documented many times before. Projects undertaken by the PsychENCODE have done much more sophisticated analyses into patterns of gene regulation across different cell-types, brain regions and neurodevelopment. I was surprised that very little comparison was made to the PsychENCODE datasets (including other epigenomic modalities - e.g. chromatin accessibility, histone modifications, etc.) beyond some simple enrichment assessments in enhancer regions. There is scope for much more work and sophisticated comparisons between datasets. As written, this is a very simplistic analysis of DMRs across regions. Work by the Geschwind group, for example has taken these types of analyses to a much higher level of sophistication. In general, there is limited recognition of other similar work being undertaken by other groups.

The primary strength of this study is the use of WGBS which has enabled the authors to assess variation in DNAm across the genome. As implemented, however, it is also a key LIMITATION of the study; each individual site is only covered by a relatively small number of sequencing reads and therefore the sensitivity to identify differences [and also the likelihood of false positives] is quite high. An n of 10 reads can - at best - only provide sensitivity to identify a difference of DNAm of 10%. Much deeper coverage is required to robustly identify variation between samples. The focus on WGBS also means that the number of samples profiled is actually very small [esp when looking at unique individuals per brain region]. The implications for the power of the study need to be recognized and formally discussed.

Line 106 - "Several samples were excluded due to genotype discordance or sample mislabelling" - I find this quite worrying given the relatively small number of tissues/individuals processed. It suggests a relatively high degree of experimental error introduced during the processing of the DNAm data that does not necessarily instil total confidence in the results. Could the authors elaborate more here on when/how these errors were introduced? Although these errors have been spotted, it is possible other problems have gone undetected.

It is NOT correct to describe the NeuN+ fraction as "neuronal". It suggests the authors have limited understanding about basic neurobiology. Although NeuN does mark many neuronal cells, certain types of neuron are not NeuN+ [e.g. Purkinje neurons]. Also, certain other cell-types can be NeuN+. Within the neuN+ fraction there will also be considerable heterogeneity of cell-types. Finally, the gating itself is not perfect, and this is certainly indicated by the FACS example presented in the supplementary material. Therefore, these nuclei should be described as 'NeuN+ enriched' and real caution should be excised in how these data are interpreted.

The representative NeuN+/- FANS image [supp fig 1] does not look particularly good, and the separation is far from perfect. Presumably the authors have selected an 'optimal' example. I would be concerned about just how pure these populations would be. Ideally, the authors would confirm using fluorescent microscopy and also some qPCR of known neuronal marker genes on RNA isolated from the nuclei populations. I think some further validation of the actual FANS preparation is critical for readers to have confidence that this aspect of the experiment has been successful - and it's a critical part of the process.

I am surprised that the authors chose not to also asses the NeuN- fraction. Neuronal cells from different regions of the brain will be more similar to each other than different cell-types within a given brain region - much of the regional differences are likely to be manifest in this cellular fraction. Other antibodies are available that enable to isolation of other neural cell populations [e.g. oligodendrocytes] - this is another major limitation about how this study has been designed.

There is no validation of any of the DMRs. Given the technical limitations related to low read coverage in WGBS, I think some confirmation of these differences is critical.

The weakest part of the study is the analysis of inter-individual variation. The sample size is totally underpowered for a mQTL study and I have no confidence in an n of 1 for the tested SNPs. I think this aspect of the manuscript would be best removed as it is almost pointless to undertake a QTL analysis on such a small set of samples. The data obtained from these analyses will be at best misleading and at worst full of false positives. Much bigger datasets exist for the analysis of QTLs in the brain, and the PsychENCODE consortium has extensive work in this area.

The analysis of VMRs is poorly described and noisy. It seems bizarre that there would be almost 5 times as many VMRs in amygdala than cortex. This - to me - seems implausible and needs confirmation and validation. Most inter-individual variation in DNA methylation is driven by genetic variation - this has been shown before. And we know that most QTL effects are common across tissues and cell-types. Therefore the vast differences between anatomically-proximal regions of the brain is very surprising.

There is little contemplation of other markers of gene regulation. In particular, sodium bisulfite treatment of DNA cannot distinguish between DNA methylation and hydroxymethylation. This is a modification that is known to be enriched in the brain. The authors have been very naïve in their approach by totally ignoring this important caveat. A search of the text shows that this is not mentioned once. In reality, this modification needs to be profiled in parallel to DNA methylation - e.g. using oxidative bisulfite conversion.

Finally, the Reviewers need to be provided with access to the raw data and analysis scripts. Without these it is impossible to check the code and validate the quality and interpretation of the analyses. I would like to have access to this in any revision.

**Authors Response**

**Point-by-point responses to the reviewers’ comments:**

**Reviewer #1 :**

Manuscript Number: GBIO-D-20-01078

Title: Human brain region-specific variably methylated regions (VMRs) are enriched for

heritability of distinct neuropsychiatric traits

I think this paper is worthy of publication. However, my main issue with the current format is that I find much of the way the data is presented rather impenetrable, and it took me multiple reads of the paper to digest it all and figure out some of the things that were being presented. I found that several of the figures are displayed in ways that make it very hard for the reader to figure out what they are actually showing. Acronyms are used very frequently, and are often completely lacking from the figure legends. At points I sometimes felt I had to learn a new language to try and understand what was being displayed when looking at the some of the figures with all the acronyms that were used... I realize what the authors have done here encompasses multiple tissues, both CpG and non-CpG methylation, and analysis of both differential (fixed) and variable (polymorphic) methylation, but in its current form it is at points so thick with acronyms that you have to think really hard about some sentences and figures to decipher what is being stated or shown. I therefore think that the authors need to work on ways of making these data a bit more accessible to the reader. I know there is a balance to be made of using acronyms to save repetition and word count, but I would like to see a revision in which they try to ensure figures can be understood as stand-alone objects without being forced to comb through the text (I think this is important as readers often look at figures without reading everything), and perhaps removing some of the acronyms used, particularly for items that are not stated very often.

*Thank you for your valuable comments regarding clarity (here and below), which we believe have helped to improve the manuscript. We have reduced the use of acronyms as much as possible and worked additionally to improve clarity throughout the manuscript and in the figures and legends.*

Specific Comments

While the methodology used to define DMRs is explained in the Methods section, I think it would be of benefit to briefly state what was done at the beginning of the Results when DMRs are first mentioned. This would give the reader some context of what these are before then talking about how many DMRs were found.

*We have improved clarity in this section to convey how DMRs were identified. We now write*

*Lines 126 onwards:” We performed a differential analysis of CpG methylation identifying CG-DMRs, i.e. regions of differential CpG methylation among neuronal nuclei isolated from each brain region. Given that a single CG-DMR can represent a difference among multiple brain regions, rather than perform 28 pairwise comparisons, we used an F-test to identify 174,482 statistically significant autosomal neuronal CG-DMRs which are defined as regions of the genome where at least 2 of the 8 brain regions have different levels of CpG methylation (****Supplemental Table S4****). We control the family-wise error rate at 5% by permutation, and we use BSmooth to leverage information from nearby CpGs by smoothing.”*

Line 127, Results: Authors state "CG-DMRs encompassed 99.5% 128 (13019/13074) of the CG-DMRs we previously identified [11]." This statement seems either unnecessary, or needs a bit more explanation. To make it clearer, if the authors here are trying to indicate that there is very strong agreement between the results in this present study and a previous one, I think it would be best to state exactly that. Otherwise, as written, this statement is rather hanging and unconnected to anything else before or after.

*This statement emphasizes the reproducibility of our methods identifying DMRs. We have made this clearer so this point comes across to the reader.*

*Lines 134-137:” In a pilot study, we profiled NeuN+ cells from 4 brain regions using whole genome bisulfite sequencing on samples from 6 different individuals not part of GTEx [12]. We replicate 99.5% of our previously identified CG-DMRs (13019/13074), highlighting the reproducibility of our experimental and analytical approaches across biobanks.”*

In the abstract, the authors state they identified conventional differentially methylated regions (DMRs)". The term DMR is used in many different contexts in the literature, e.g. to notate regions that have differential methylation between samples or cohorts (e.g. case vs control studies), in relation to imprinted loci (i.e. differential methylated between parental alleles), or what I suspect the authors mean here, which is differential methylation among tissues. I think they should state clearly what the "differential" of "differential methylation" is referring to when used in this manuscript.

*Indeed, the DMR terminology is often confusing. We have specified the meaning of DMRs in the abstract and introduction.*

*Lines 38-41 (abstract): “We identified differentially methylated regions between brain regions (DMRs) among neuronal nuclei in both CpG (181,146) and non-CpG*

*(264,868) contexts, few of which were unique to a single pair-wise comparison.”*

Similarly in the introduction, it is stated "enabled our identification of variably methylated regions (VMRs)". Again, this term is used without definition, which I think is key to enable most readers to understand clearly what the study is doing. Here VMR is used by the authors to refer to regions of variable methylation observed within a single tissue among different individuals (ie inter-individual polymorphism), but this needs to be explicitly defined when first talked about.

*We have more clearly defined what we mean by VMRs in the text.*

*Line 45-48:”We also identified 81,130 regions of variably CpG methylated regions (VMRs), i.e. variable methylation among individuals in the same brain region, which were enriched in regulatory regions and in CpG DMRs.”*

*We have a more extensive description in the Results section.*

Results: The authors state "we generated >30B uniquely mapped 150bp paired end reads". Instead of B, better to say billion?

*We have made this edit.*

Table 2 displays the fraction of VMRs that overlap SNPs of varying allele frequencies. This seems not a very useful result to me, as this is a very crude measure, and it's not quite clear what the point of this table is. My guess is the authors are trying to show that ubiquitous VMRs have a slightly increased fraction of common SNPs, but this is never commented on, and not formalized in any way (is it significant?). Even if this is the point and it is significant, I think it would be better simply stated in the text, and does not require a dedicated table in the manuscript (maybe Supplementary would be better?). To hammer this home, the authors then go on to identify SNPs associated with VMRs, and look +/-50kb around (not just overlapping), indeed finding many associated SNPs which lie outside the VMRs. Thus, the relevance of SNPs directly overlapping VMRs is diminished.

*We were not clear with regard to the reasoning behind this analysis. Our thinking is as follows: for expression, it is clear how a SNP could be associated with the expression of a nearby gene by (for example) modulating protein binding or other features of the regulatory regions (promoter, enhancer) of the gene. This type of mechanism is not so clear for DNA methylation. Indeed, the most obvious way that a SNP could be (causally and directly) associated with DNA methylation in a specific region is through a very local mechanism, for example by changing DNA-protein binding inside the region. For this reason, we were quite interested in the distribution of SNPs inside VMRs. Because we have whole-genome DNA sequencing (and not just genotyping arrays) we can assess the overlap well.*

*However, our analysis does not yield any clear conclusion with regard to this question. We have therefore moved the table to the supplement (although we note that Genome Biology largely prefers small tables to be in the manuscript due to its online nature).*

I find Figures 1c and 1d rather confusing (especially 1d). CG-blocks are not explained/defined, and there is only one very brief mention of them in the text of the Results, so it is not clear to me what these are exactly (I'm guessing large chunks of differential methylation?). I think part of the issue is the notation. What is HC, BG and All? I eventually found them listed in the legend, but why not just write hippocampal and basal ganglia in the plot? The color scale could be much better labeled, as "log2OR" is not clear. Why not say "log2 enrichment" (I presume OR=Odds Ratio?)? But in 1d I am also confused as to how there can be HC (I think this means Hippocampal) on the x axis, but then the y-axis also includes Hippocampus?

*We performed three different analyses to identify DMRs among the 5 tissues groups (All), among just the basal ganglia brain regions (BG) and between the 2 hippocampus groups identified by PCA (HC). We have removed tissue abbreviations as suggested in this and all other figures where possible and made other changes to enhance figure clarity. We will relabel our color scales as recommended as you are correct in that OR stands for odds ratio. For 1d, we are showing enrichments of our data (noted across bottom) in ATAC data from the different brain regions (including hippocampus) from Fullard 2018 across the right side and this has been made clearer in the figure. The word “blocks” – which stems from our terminology in previous work – has been clarified; it is indeed large regions of differential methylation, but defined a little differently from DMRs (we use a larger smoothing bandwidth which removes small differences).*

Fig1d shows "OCRs', but these are not explained. I managed to track this down to be "Open Chromatin Regions", but OCR is never defined anywhere in the entire manuscript as far as I could see.

*We have defined OCRs in the text and figure legend to improve clarity and removed this abbreviation as it was not used frequently.*

Fig2A. The format used to show the color/study is confusing. Why not just use 3 different symbols rather than a combination of color and shape, which I found hard to disentangle. Also surely there is a better notation than "NatNeuro", which is very generic and [ambiguous. eg](http://ambiguous.eg). "Rizzardi et al 2019"?

*We have adjusted the figure as recommended to improve clarity and replaced “NatNeuro” with the correct citation. That was internal terminology and it was a mistake to have kept it in.*

Figure 5: This figure shows "BG CG DMRs" and "CG DMRs". Although they do define in the text, neither are defined (or even mentioned) in the figure legend. It would be better to not use an acronym for BC here. If someone simply looks at the figures, it is impossible to understand what is being shown without first digging through the text to track down where these are defined.

*We have spelled out this acronym to increase clarity throughout the manuscript and figures/legends.*

Figure 6 has way too many acronyms, none of which are explained to the reader. The x-axis of Fig 6c has reduced different cell types (I think?) to 2 or 3 letter codes, but these are not at all clear. Furthermore there are other things listed such as nVMRs - what is an nVMR, and how is it different to a VMR? What is a ubiquitous VMR? I don't see what the left heat map panel adds, as it should be obvious that nVMRs overlap a lot with All VMRs or ubiquitous VMRs. Then I am also not clear what is the difference between the other two panels, which are labeled "Tissue Specific VMRs" and "All VMRs per tissue"? I not sure I understand what is being presented here.

*These letter codes used throughout the paper have now been spelled out and include the relevant reference when applicable to enhance clarity and to be clear what data are being compared. In 6c, we are comparing our VMRs to those identified in two other studies using different criteria and showing that we are capturing much of the same information, but adding additional tissue-specific information that was lacking in those studies as they were looking for variable methylation that was shared among all tissues tested (similar to our “ubiquitous VMRs” which are shared among all tissues we tested). The left heatmap shows that the enrichment is highest for ubiquitous VMRs and mQTL VMRs compared to the union of All VMRs or any tissue-specific VMRs. This part of the figure serves to distinguish our VMRs from those previously reported by other groups. We have explicitly defined “ubiquitous VMRs” in all figure legends and in the text to improve clarity.*

**Reviewer #2 :**

This study uses samples from GTEx to assess differences in DNA methylation between brain regions and individuals. Although tackling an important question, overall the analyses presented in this manuscript are not particularly novel or ground-breaking. It is not surprising that DNA methylation varies across different regions (and cell-types) of the human brain - this has been documented many times before. Projects undertaken by the PsychENCODE have done much more sophisticated analyses into patterns of gene regulation across different cell-types, brain regions and neurodevelopment. I was surprised that very little comparison was made to the PsychENCODE datasets (including other epigenomic modalities - e.g. chromatin accessibility, histone modifications, etc.) beyond some simple enrichment assessments in enhancer regions. There is scope for much more work and sophisticated comparisons between datasets. As written, this is a very simplistic analysis of DMRs across regions. Work by the Geschwind group, for example has taken these types of analyses to a much higher level of sophistication. In general, there is limited recognition of other similar work being undertaken by other groups.

*The reviewer is correct that we have not cited and discussed psychENCODE. We do disagree with the suggestion that we are not recognizing similar work (DNA methylation in brain) by other groups though.*

*We have now delved deeper into DNA methylation and psychENCODE, something that is not entirely easy to understand. The most relevant papers are – as far as we can ascertain – Wang et al [1], Li et al [2] and Kozlenkov et al [3]. These three papers – despite all being from psychENCODE – use non-overlapping datasets.*

*Wang et al [1] – in our reading the most relevant paper to our work – uses the 450k array in 269 samples (about 20 individuals) across 16 brain regions throughout development. However, they did no sorting on these tissues and our previous work [4] established (by a clear apples to apples comparison on the same individuals) that the majority of tissue-specific differences in methylation will be missed in bulk tissue analyses. In addition, this study did not discriminate among the striatal regions (nucleus accumbens, caudate, putamen) whereas our study is the first to identify substantial differential methylation among neurons isolated from these three structures. So the advantages of our study are numerous (1) NeuN+ sorting vs bulk (2) WGBS vs 450k (3) at least same sample size (depending on brain region), especially because we do not cover the same age range (4) much better coverage of the striatal regions.*

*Li et al [2] – probably the landmark psychENCODE paper – does not produce their own DNA methylation data. Instead they reuse 450k data from Jaffe et al [5], which profiles ~500 bulk samples across many developmental time points but only on the prefrontal cortex (controls and disease); only 225 of which are used by psychENCODE. With only 1 brain region and 450k data on bulk samples, this is a much less comprehensive study than ours.*

*Kozlenkov et al [3] uses WGBS together with oxBS (for hydroxymethylation) on more refined cell populations (3 populations: glutamatergic excitatory neurons, medial ganglionic eminence– derived γ-aminobutyric acid (GABA)ergic inhibitory neurons, and oligodendrocytes). However, all the methylation samples are from only 2(!) individuals and the study profiles 3 brain regions (prefrontal cortex, cerebellum and temporal cortex). Our study has a much larger sample size, and is much more diverse with regard to brain regions – but does not cover the same breadth of assays and is less cell population specific.*

*In our opinion, it is pretty clear from these numbers that our study substantially advances the available DNA methylation data across the human brain, especially in NeuN+ cells. As we have established in our pilot study [4], CG-DMRs in NeuN+ cells carry much more heritability for neuropsychiatric diseases compared to CG-DMRs in NeuN- cells as well as regions defined using ATAC-seq, which is the basis for our focus on this cell population and this epigenetic modality. However, there is no doubt that psychENCODE is a much richer data integration analysis across epigenetic marks and RNA. We have now included these references in the manuscript and acknowledged the important contributions of PsychENCODE:*

*Lines 64-71:”The PsychENCODE consortium has made substantial contributions to our understanding of gene regulation in the human brain, particularly during development and disease [13, 14], and in distinct cell populations within the cortex [15]. However, these efforts have been somewhat limited either by examination of multiple cell populations within a single cortical region or by the use of bulk tissues that are strongly confounded by cellular heterogeneity. Thus, the extent of DNA methylation variation within neuronal populations from many adult human brain regions remains unknown and is the focus of this work.”*

1. *Li M, Santpere G, Imamura Kawasawa Y, Evgrafov OV, Gulden FO, Pochareddy S, Sunkin SM, Li Z, Shin Y, Zhu Y, et al. 2018. Integrative functional genomic analysis of human brain development and neuropsychiatric risks. Science* ***362****: eaat7615-15.*
2. *Wang D, Liu S, Warrell J, Won H, Shi X, Navarro FCP, Clarke D, Gu M, Emani P, Yang YT, et al. 2018. Comprehensive functional genomic resource and integrative model for the human brain. Science* ***362****.*
3. *Kozlenkov A, Li J, Apontes P, Hurd YL, Byne WM, Koonin EV, Wegner M, Mukamel EA, Dracheva S. 2018. A unique role for DNA (hydroxy)methylation in epigenetic regulation of human inhibitory neurons. Science Advances* ***4****: eaau6190*
4. *Rizzardi LF, Hickey PF, Rodriguez DiBlasi V, Tryggvadóttir R, Callahan CM, Idrizi A, Hansen KD, Feinberg AP. 2019. Neuronal brain-region-specific DNA methylation and chromatin accessibility are associated with neuropsychiatric trait heritability. Nature Neuroscience.*
5. *Jaffe AE, Gao Y, Deep-Soboslay A, Tao R, Hyde TM, Weinberger DR, Kleinman JE. 2016. Mapping DNA methylation across development, genotype and schizophrenia in the human frontal cortex. Nat Neurosci* ***19****: 40–7.*

The primary strength of this study is the use of WGBS which has enabled the authors to assess variation in DNAm across the genome. As implemented, however, it is also a key LIMITATION of the study; each individual site is only covered by a relatively small number of sequencing reads and therefore the sensitivity to identify differences [and also the likelihood of false positives] is quite high. An n of 10 reads can - at best - only provide sensitivity to identify a difference of DNAm of 10%. Much deeper coverage is required to robustly identify variation between samples. The focus on WGBS also means that the number of samples profiled is actually very small [esp when looking at unique individuals per brain region]. The implications for the power of the study need to be recognized and formally discussed.

*Respectfully, we have been using our smoothing approach since we pioneered it in 2011[1,2], published in this journal. We – and others - have validated it multiple times. Our approach is to use the well-known correlation between nearby CpGs to improve coverage. Importantly, a study in Nature Methods [3] showed that – for a fixed sequencing budget – it is better to assay more samples at lower coverage than fewer samples at higher coverage, which is exactly the strategy we have employed. We have used these findings extensively when we designed our study. There is no doubt that given unlimited resources, we would rather haver deeper coverage on the same individuals, but that is also substantially more expensive.*

*But don’t take our word for it. In our present manuscript, we compare our reported eGTEx data to our previous study [4], which was in fact a pilot study for our present – much larger - experiment. This pilot study was performed on other individuals (from the Maryland brain bank), but was otherwise identical. We replicate 99.5% of the DMRs identified in our pilot study, which is rather remarkable. The fact that we replicate the DMRs does not of course mean that they are functional, but this is still a rather impressive replication rate, given the samples are from two different Biobanks. This fact was not sufficiently highlighted in the first version of our manuscript and we thank the reviewer for drawing attention to this.*

1. *Hansen KD, Timp W, Bravo HC, Sabunciyan S, Langmead B, McDonald OG, Wen B, Wu H, Liu Y, Diep D, et al. 2011. Increased methylation variation in epigenetic domains across cancer types. Nat Genet* ***43****: 768–75.*
2. *Hansen KD, Langmead B, Irizarry RA. 2012. BSmooth: from whole genome bisulfite sequencing reads to differentially methylated regions. Genome Biol* ***13****: R83.*
3. *Ziller MJ, Hansen KD, Meissner A, Aryee MJ. 2015. Coverage recommendations for methylation analysis by whole-genome bisulfite sequencing. Nat Methods* ***12****: 230–2.*
4. *Rizzardi LF, Hickey PF, Rodriguez DiBlasi V, Tryggvadóttir R, Callahan CM, Idrizi A, Hansen KD, Feinberg AP. 2019. Neuronal brain-region-specific DNA methylation and chromatin accessibility are associated with neuropsychiatric trait heritability. Nature Neuroscience.*

Line 106 - "Several samples were excluded due to genotype discordance or sample mislabelling" - I find this quite worrying given the relatively small number of tissues/individuals processed. It suggests a relatively high degree of experimental error introduced during the processing of the DNAm data that does not necessarily instil total confidence in the results. Could the authors elaborate more here on when/how these errors were introduced? Although these errors have been spotted, it is possible other problems have gone undetected.

*We described this poorly in our first submission. What we are reporting on is a discrepancy between the samples sent to us by the Biobank and the internal records of the biobank that we (and others) reported to the consortium. The GTEx biobank has around 30,000 tissue samples. Furthermore, the GTEx brain samples were dissected in Miami by the Miami brain bank unlike the other GTEx samples. This opens up plenty of possibility for sample mislabeling at the biobank level. Using PCA, we found five samples that clustered with the “wrong” tissue based on their metadata from the tissue bank. We compared the methylation of these samples to the average methylation of each brain region to identify these samples (Fig.S1b). These samples were also excluded because even after detailed communication with the tissue bank we could not confirm the tissue of origin by any other metric. In addition, for lung and thyroid we received DNA (not tissue) in a 96-well plate where several wells had been overfilled. Which is likely to source of contamination resulting in failed genotyping QC for the non-brain samples. We have no QC failures on our methylation data, only on sample identity prior to receipt by our lab. We now write:*

*Lines 119-123: “Several samples were excluded due to genotype discordance, as the shipped sample genotype did not match the biobank records (****Supplemental Table S3****). Five additional samples were excluded after principal component analysis revealed sample mislabeling prior to receipt by our lab. We confirmed this by determining which tissues most closely matched the methylation of these samples (****Fig. S1b****).”*

It is NOT correct to describe the NeuN+ fraction as "neuronal". It suggests the authors have limited understanding about basic neurobiology. Although NeuN does mark many neuronal cells, certain types of neuron are not NeuN+ [e.g. Purkinje neurons]. Also, certain other cell-types can be NeuN+.

*We are well aware of the properties of the NeuN antibody and it has been widely used to segregate neuronal from non-neuronal nuclei from post-mortem brain for at least a decade. We are aware that some neuronal populations are not detected with this antibody, but none of those populations, listed below, are present in any of the brain structures we have profiled in this study: cerebellar Purkinje cells and dentate nucleus neurons, Cajal*–*Retzius cells in developing cerebellum, olfactory bulb mitral cells, retina photoreceptor cells, brain stem dorsal cochlear nucleus neurons, and sympathetic chain ganglion neurons along the spinal cord. However, NeuN is not present in other cell types as it is expressed exclusively in neuronal tissues. There are reports of* in vitro *staining of NeuN+/GFAP+ astrocytes, but this only occurs in certain culture systems and is not seen in mouse or human tissues. Interestingly, a fibroblast cell line (3T3) also stains positive for NeuN [1]. Finally, we might add that the psychENCODE paper [2] uses the same “neuronal” terminology for NeuN+ cells (although not to the extent that we do).*

1. *Gusel’nikova VV, Korzhevskiy DE. 2015. NeuN As a Neuronal Nuclear Antigen and Neuron Differentiation Marker. Acta Naturae* ***7****: 42–47.*
2. *Li M, Santpere G, Imamura Kawasawa Y, Evgrafov OV, Gulden FO, Pochareddy S, Sunkin SM, Li Z, Shin Y, Zhu Y, et al. 2018. Integrative functional genomic analysis of human brain development and neuropsychiatric risks. Science* ***362****: eaat7615-15*

Within the neuN+ fraction there will also be considerable heterogeneity of cell-types.

*We are aware of this, however, the largest source of confounding is removed when you separate NeuN+ from NeuN- and we have shown that you can detect substantial differences in*

*methylation among NeuN+ nuclei isolated from functionally distinct brain regions both in this work and our previous publication [1]. So, we can detect functional methylation differences between the neuronal populations from one brain region compared to another. We agree more separation among neuronal subtypes is highly desirable, but this is not feasible to do across many brain regions as even if it were possible, not all subtypes are present in all brain regions preventing us from asking our main question: how does methylation differ in neurons across the brain. We are aware of Stella Dracheva’s work [2] using Sox6 to separate excitatory from inhibitory neurons, however, this subdivision will not isolate the exact same populations of neurons when applied to different brain regions. Single cell analyses could be used, however, sample size is still a limitation and single cell methylation data are not yet as comprehensive or robust as other single cell data types.*

*Finally, our pilot data [1] strongly suggests that at the level of the NeuN- cell population, there is no methylation differences between brain regions in adult – normal – brain. Or at least we found no differences in the 4 brain regions tested in [1]. This was btw. confirmed by both RNA-seq and ATAC-seq on the same samples on sorted cells (NeuN +/-). This is in contrast to the massive differences in the NeuN+ cells. There are important caveats to this: (1) this does not say anything about disease and (2) this does not prevent brain region differences in more refined sub populations of NeuN- cells. Nevertheless, we chose to focus on NeuN+ cells.*

*1. Rizzardi LF, Hickey PF, Rodriguez DiBlasi V, Tryggvadóttir R, Callahan CM, Idrizi A, Hansen KD, Feinberg AP. 2019. Neuronal brain-region-specific*

*DNA methylation and chromatin accessibility are associated with neuropsychiatric trait heritability. Nature Neuroscience.*

*2. Kozlenkov A, Wang M, Roussos P, Rudchenko S, Barbu M, Bibikova M, Klotzle B, Dwork AJ, Zhang B, Hurd YL, et al. 2016. Substantial DNA methylation differences between two major neuronal subtypes in human brain. Nucleic Acids Res* ***44****. 2593–612.*

Finally, the gating itself is not perfect, and this is certainly indicated by the FACS example presented in the supplementary material. Therefore, these nuclei should be described as 'NeuN+ enriched' and real caution should be excised in how these data are interpreted.

The representative NeuN+/- FANS image [supp fig 1] does not look particularly good, and the separation is far from perfect. Presumably the authors have selected an 'optimal' example. I would be concerned about just how pure these populations would be. Ideally, the authors would confirm using fluorescent microscopy and also some qPCR of known neuronal marker genes on RNA isolated from the nuclei populations. I think some further validation of the actual FANS preparation is critical for readers to have confidence that this aspect of the experiment has been successful - and it's a critical part of the process.

*We strongly disagree with the reviewer’s assessment of our sorting accuracy. There are several reasons the reviewer could be concerned. One is the inclusion of a panel showing the gating out of autofluorescence (FigS1A, bottom left, this paper) which most authors don’t include. Another is us showing the full plot of our DAPI x NeuN-488 (FigS1A bottom right, this paper) where you can see some degraded nuclei that are less DAPI positive and are gated out along with some 4N nuclei that show increased DAPI staining. This occurs in other’s samples as well, but could be cut off in the plots they show. Another reason it might not appear is that many authors show a subsample of their sort data, usually 10,000 events. We have shown as much larger number of events for completeness (>100k). This can make the small % of nuclei not in selected populations look more pronounced than they really are compared to the populations of interest. Further, the same sample preparation methods and antibodies can look somewhat different across different sorters depending on the software or who is setting up the gates. We sorted on a MoFlo (our supp fig) and it looks somewhat different than on a Sony MA900 for example (my own data, black background above, only 10,000 events). The samples were processed exactly the same and they are both cortical tissue samples, yet they do look a bit different.*

*In addition, our data look very similar to data produced by the Akbarian lab who refined this protocol for sorting nuclei from post-mortem brain tissues. See Figure S19 from [1] copied to the left. Further, post-sort analysis of our data showed that our samples were >95% pure NeuN+ or NeuN- as appropriate.*

1.	*Girdhar K, Hoffman GE, Jiang Y, Brown L, Kundakovic M, Hauberg ME, Francoeur NJ, Wang Y, Shah H, Kavanagh DH, et al. 2018. Cell-specific histone modification maps link schizophrenia risk to the neuronal epigenome. Nat Neurosci* ***21****: 1126–1136*.

I am surprised that the authors chose not to also asses the NeuN- fraction. Neuronal cells from different regions of the brain will be more similar to each other than different cell-types within a given brain region - much of the regional differences are likely to be manifest in this cellular fraction. Other antibodies are available that enable to isolation of other neural cell populations [e.g. oligodendrocytes] - this is another major limitation about how this study has been designed.

*The reviewer is correct that there are substantial methylation differences between NeuN+ and NeuN- populations and we and others have reported this previously. However, our previous work (Rizzardi et al, 2018) also showed that regional methylation differences were restricted to the NeuN+ fraction as we found >13k DMRs among NeuN+ samples from 4 brain regions compared to only 114 in the NeuN- fraction. Therefore, we purposefully chose to analyze the cellular fraction that yielded the most information about regional differences. Given unlimited resources, we agree that profiling other more specific populations would have yielded valuable insights, however, the goal of GTEx is to profile as many individuals as possible and we chose to focus on the NeuN+ fraction in as many individuals as our budget allowed.*

There is no validation of any of the DMRs. Given the technical limitations related to low read coverage in WGBS, I think some confirmation of these differences is critical.

*We respectfully disagree as we have already addressed the reviewer’s concern regarding low coverage and we do replicate 99.5% of the DMRs identified in Rizzardi et al, 2018 in our current study. The exact same methods were used in both studies though the tissues were acquired from two different sources. This represents a remarkable level of replication that we have now emphasized in the manuscript.*

*Lines 134-137: “In a pilot study, we profiled NeuN+ cells from 4 brain regions using whole genome bisulfite sequencing on samples from 6 different individuals not part of GTEx [12]. We replicate 99.5% of our previously identified CG-DMRs (13019/13074), highlighting the reproducibility of our experimental and analytical approaches across biobanks.”*

The weakest part of the study is the analysis of inter-individual variation. The sample size is totally underpowered for a mQTL study and I have no confidence in an n of 1 for the tested SNPs. I think this aspect of the manuscript would be best removed as it is almost pointless to undertake a QTL analysis on such a small set of samples. The data obtained from these analyses will be at best misleading and at worst full of false positives. Much bigger datasets exist for the analysis of QTLs in the brain, and the PsychENCODE consortium has extensive work in this area.

*Our mQTL analysis is clearly underpowered. We say so explicitly in the Results, and we don’t even mention this analysis in our abstract. We therefore believe we are very clear with regard to the limitations of this analysis. However, we disagree with the reviewer on whether this part of the analysis is useless. We note that it is easy to find molecular QTL studies using smaller sample sizes than we are using including all initial reports of QTLs for histone modifications and 3D structure*. *As detailed above, the psychENCODE DNA methylation data has a comparable (or smaller) sample size as ours (while being in bulk tissue and using 450k arrays), apart from their re-use of an earlier dataset in prefrontal cortex.*

The analysis of \TMRs is poorly described and noisy. It seems bizarre that there would be almost 5 times as many \TMRs in amygdala than cortex. This - to me - seems implausible and needs confirmation and validation. Most inter-individual variation in DNA methylation is driven by genetic variation - this has been shown before. And we know that most QTL effects are common across tissues and cell-types. Therefore the vast differences between anatomically-proximal regions of the brain is very surprising.

*We were also surprised by the large amounts of VMRs in amygdala. Which is why we did further analysis (reported in lines 281-306), which strongly supports our hypothesis that this is due to confounding by sub region. Or in other words, different samples from different individuals come from different sub regions of the amygdala. This is for example supported by a comparison to single-cell RNA data from amygdala, where we identify VMRs within 1kb of many human homologues of amygdala-sub region specific genes in mouse.*

*With regard to the contribution from genetics, we believe our study is a substantial contribution to this question. Most existing studies only profile samples from a single brain region and many do not use sorted cells. Our data suggests that most VMRs are not shared across all brain regions. This does not mean that the VMRs are not genetically driven of course, but it means that methylation is only driven by genetics in some – but not all – brain regions.*

There is little contemplation of other markers of gene regulation. In particular, sodium bisulfite

treatment of DNA cannot distinguish between DNA methylation and hydroxymethylation. This is a modification that is known to be enriched in the brain. The authors have been very naïve in their approach by totally ignoring this important caveat. A search of the text shows that this is not mentioned once. In reality, this modification needs to be profiled in parallel to DNA methylation - e.g. using oxidative bisulfite conversion.

*Given unlimited resources we – of course – want to profile as many markers as possible. However, that is not what we chose to do, with our limited resources. As the reviewer points out, WGBS measures the combination of CpG methylation and hydroxymethylation. However, the kits available for measuring hydroxymethylation (including oxBS and TAB-seq) have never been successfully used in large sample studies. A recent study (Kozlenkov et al, 2018 Sci Adv) utilized oxBS, but only profiled sorted nuclei from the prefrontal cortex of 2 individuals. It is highly non­trivial to get good data on this modification. We know this, we published one of the first analyses of hydroxymethylation in human cancers (Li et al 2015 Genome Res). In addition, profiling hydroxymethylation requires substantially deeper WGBS (for technical reasons) meaning that adding hydroxymethylation to our study could easily increase the total cost by 3x or more. Finally, GTEx tissue was limiting as it was shared across many groups and at the time we were processing these samples, input requirements for oxBS were prohibitive (>2 ug DNA).*

*While discussing brain specific epigenetic marks, the reviewer must be delighted that we have the – by far – best and most comprehensive analysis of non-CpG methylation which is also brain specific, and is not possible to analyze using Illumina methylation arrays.*

*In our writing we unfortunately assumed that everyone already knows this, but clearly our work is interesting to a large body of researchers interested in the brain, but without the background in epigenetics to know these things. We have therefore added the following to discussion:*

*Lines 392-396: “Bisulfite sequencing does not distinguish between cytosine methylation and hydroxymethylation. The human brain has elevated levels of hydroxymethylation (~10-15%) compared to other tissues [53, 54]. In neuronal subpopulations isolated from adult prefrontal cortex, 5hmC levels can be as high as 40% [15], and it will be important in future studies to accurately profile this mark across different brain regions.”*

Finally, the Reviewers need to be provided with access to the raw data and analysis scripts. Without these it is impossible to check the code and validate the quality and interpretation of the analyses. I would like to have access to this in any revision.

*The authors provided links to the data and code used in this study in the “Availability of data and materials” section, Lines 707-714. The protected data will be part of the GTEx v9 dbGaP release and should be available within two weeks. However, we are unable to provide a review link to this protected data. The open-access data will be publicly available on the GTEx Portal and includes visualizations of aggregated methylation values for each tissue and the DMR/VMR tables provided with this manuscript, similar to what we provided on UCSC for our previous work (*[*http://genome.ucsc.edu/cgi-bin/hgTracks?db=hg19&hubUrl=https://s3.us-east-2.amazonaws.com/brainepigenome/hub.txt*](http://genome.ucsc.edu/cgi-bin/hgTracks?db=hg19&hubUrl=https://s3.us-east-2.amazonaws.com/brainepigenome/hub.txt).)*).*

**Second round of review**

**Reviewer 1**

I think the data presented is now much easier for the reader to understand than the original version, thanks!

**Reviewer 2**

The authors provide a lengthy rebuttal to my major concerns without actually trying to address most of the comments suggested. Therefore, I still have some major reservations about many of the analyses. I still think these could be addressed if the authors were willing to do a little extra work. Also, they still have not provided full access to raw data which makes checking the paper fully very difficult for reviewers. For an article this complicated, this is critical.

1. "Respectfully, we have been using our smoothing approach since we pioneered it in 2011…there is no doubt that given unlimited resources, we would rather haver deeper coverage on the same individuals, but that is also substantially more expensive…In our present manuscript, we compare our reported eGTEx data to our previous study[4], which was in fact a pilot study for our present -much larger -experiment... We replicate 99.5% of the DMRs identified in our pilot study, which is rather remarkable." With respect to the authors, this data is not shown at all in the manuscript so it is very hard to gauge how accurate this claim of replication is. How can readers interpret this statement? I would appreciate seeing the actualities of this comparison and understanding exactly what they mean by replication here [e.g. does it mean significant multiple-testing-corrected P-value?]. Please add this to any revision.

2. While covering more CpG sites, respectfully there is no way that WGBS can provide the sensitivity of the Illumina arrays at the depth of sequencing the authors have employed. OK, they can estimate values in proximal sites using correlation, but this is not perfect especially without a very detailed ultra-deep reference to refer the data to. One way to convince me and other readers would be to validate a number of differences using more sensitive targeted approaches. These are quick, cheap and easy to do. Or give more specific details about what they claim to be replication [as requested above]. At the moment, these claims are based on words but no data/results.

3. I am still concerned by the level of sample mix-ups in the dataset. The authors have been able to resolve those that are straightforward to do [e.g. wrong tissue] but it doesn't mean that other problems have gone unnoticed. Given the relatively small sample size [compared to genetic studies for example] this is a high level of mix-ups that warrants a bit more discussion and checking.

4. I still debate the unequivocal assumption that NeuN+ = neuronal. There is much evidence that this is not always the case. E.g. https://www.ncbi.nlm.nih.gov/pubmed/17995928, and I think the jury is still out about exactly how specific NeuN is. Perhaps simply say "neuron-enriched"? The authors words imply that sorting neun+ nuclei is a panacea. It is still a very broad population of cell-types and there will be considerable heterogeneity. It is highly plausible that specific neuronal cell populations are affected in schizophrenia - there is a huge literature on this. So it is not correct to assume that any between-individual heterogeneity in cell proportions is removed by doing this step - it's a very naïve assertion. In fact, some more refined cell-type estimates can be derived using some of the data from specific neuronal populations performed by several of the psychencode groups. I think this needs more work and more discussion.

5. Re the efficiency of the FANS method, I still think this needs validation. Without getting too defensive, the authors could do this easily by applying a statistical deconvolution method such as CETS to their data. Hopefully the neuronal estimates from their data are 100%. Or they could look at DNAm in promoter regions of some key neuron-expressed genes.

6. I still strongly believe that the mQTL section should be removed. It really is very underpowered and prone to false positives. There is no way this can be construed as a robust analysis given the number of samples included in the genotype groups. The fact that the authors themselves note that they didn't mention it in the abstract highlights how they feel this is also exploratory.

7. "Our data suggests that most VMRs arenot shared across all brain regions. This does not mean that the VMRs are not genetically driven of course, but it means that methylation is only driven by genetics in some -but not all -brain regions". This is a very surprising result and remains so. The default has to be that there is some technical issue as major inter-individual variation generally results from genetic factors and is largely correlated across cells/tissues. It is known from repeated studies in the literature that genetically-driven DNA methylation variation is correlated across brain regions (and also between brain and periphery]. This section might benefit from an interpretation in the context of the work by Rob Waterland - e.g. see https://genomebiology.biomedcentral.com/articles/10.1186/s13059-019-1708-1

8. "However, the kits available or measuring hydroxymethylation (including oxBS and TAB-seq) have never been successfully used in large sample studies." This is not true. There are several studies that have used the OxBS approach in large numbers of samples, including in brain - e.g. https://www.ncbi.nlm.nih.gov/pmc/articles/PMC5604137/

9. "However, we are unable to provide a review link to this protected data." Why not? Without having access to this to check the analyses it is not [possible to fully review the paper. This goes against the submission standards expected in Genome Biology…

**Authors Response**

**Point-by-point responses to the reviewers’ comments:**

**REVIEWER #1**

I think the data presented is now much easier for the reader to understand than the original version, thanks!

*Thank you.*

**REVIEWER #2**

The authors provide a lengthy rebuttal to my major concerns without actually trying to address most of the comments suggested. Therefore, I still have some major reservations about many of the analyses. I still think these could be addressed if the authors were willing to do a little extra work. Also, they still have not provided full access to raw data which makes checking the paper fully very difficult for reviewers. For an article this complicated, this is critical.

1. "Respectfully, we have been using our smoothing approach since we pioneered it in 2011...there is no doubt that given unlimited resources, we would rather have deeper coverage on the same individuals, but that is also substantially more expensive...In our present manuscript, we compare our reported eGTEx dta to our previous study[4], which was in fact a pilot study for our present -much larger -experiment... We replicate 99.5% of the DMRs identified in our pilot study, which is rather remarkable." With respect to the authors, this data is not shown at all in the manuscript so it is very hard to gauge how accurate this claim of replication is. How can readers interpret this statement? I would appreciate seeing the actualities of this comparison and understanding exactly what they mean by replication here [e.g. does it mean significant multiple-testing-corrected P-value?]. Please add this to any revision.

*Re-reading our wording, we were too brief in describing our unusually strong replication result. We are saying that we applied the exact same methods to new GTEx samples as we did to samples from a different brain bank for which we identified DMRs (published in Rizzardi et al 2019) and recovered 99.5% of the same statistically significant DMRs in the new analysis. It is simply an overlap of the statistically significant regions from our original publication with the statistically significant regions identified in this study. So, all the DMRs from both sample sets had a family-wise error rate (FWER) <0.05 as determined by permutation testing – ie. both sets of DMRs were corrected for multiple testing. This overlap analysis does not consider whether the boundaries of the DMRs are the same*

*But this is just an overlap. To examine whether effect sizes replicate, we computed the mean difference between NeuN+ nuclei isolated from nucleus accumbens and frontal cortex samples in each of the two studies, over the DMR coordinates from Rizzardi et al 2019 (lifted over to hg38 from hg19). The result is unusually strong as displayed in Figure S1c and reproduced here: we have a very high degree of replication of the effect size and its direction between the two studies.*

*In fact, it is reasonable to wonder why this replication is so good. The answer is our choice of family-wise error rate which is a rather conservative error rate controlling the absolute number of errors (as opposed to the relative number of errors used by the false discovery rate (FDR)). Now, why did we choose nucleus accumbens and frontal cortex for this plot? If you read our previous study, you will see we profiled 4 brain regions, but 3 of them had similar methylation profiles. We choose frontal cortex as a representative of the 3 similar brain regions. We also – in Figure S1d and reproduced here – consider the same plot but between 2 of the brain regions with more modest differences (frontal cortex and anterior cingulate cortex). Again, our replicability is unusually high. Indeed, this plot suggests again that we might have been too conservative, although we note that the plot hides variability. It seems that we can potentially replicate DMRs with less than 10% mean difference.*

*Together, this is a highly unusual level of replication – amongst the best levels of replication we have eve seen in a genome-wide study.*

*Our revised main text describing this analysis is as follows:*

*Lines 135-146: We find that 99.5% of our previously identified neuronal CG-DMRs (13019/13074) overlap with CG-DMRs from our new analysis of GTEx samples (after correcting for multiple testing). To make a more precise comparison, we examined the correlation between the methylation differences between two tissues as measured separately in Rizzardi et al [12] and this study (tissues were nucleus accumbens and prefrontal cortex, selected because most of the CG-DMRs from Rizzardi et. al. [12]were between those two tissues). We find a striking correlation of 0.97 as shown in Figure S1c highlighting the reproducibility of our experimental and analytical approaches across biobanks. This high level of reproducibility holds even when examining methylation differences among all 13,074 neuronal DMRs identified in [12] between two very similar cortical regions (Figure S1d), which suggests that our approach is conservative, likely because we control the family-wise error rate.*

2. While covering more CpG sites, respectfully there is no way that WGBS can provide the sensitivity of the Illumina arrays at the depth of sequencing the authors have employed. OK, they can estimate values in proximal sites using correlation, but this is not perfect especially without a very detailed ultra-deep reference to refer the data to. One way to convince me and other readers would be to validate a number of differences using more sensitive targeted approaches. These are quick, cheap and easy to do. Or give more specific details about what they claim to be replication [as requested above]. At the moment, these claims are based on words but no data/results.

*We agree that the Illumina methylation arrays are more precise when measuring a single CpG. But the focus in our work – and much of the functional work of the effect of DNA methylation – is on regional methylation. And for that there is substantial power by considering many nearby CpGs. There is also robustness – we would argue that sequencing is less likely to be affected by genetic variants causing a technical issue (as when the genetic variant is a CpG to TpG SNP) – although in extreme cases this can still affect WGBS as we recently studied in Wulfridge et al (2019 NAR).*

*Having said all of this, we challenge the reviewer to find an array-based study with the same level of replication as we have shown here.*

3. I am still concerned by the level of sample mix-ups in the dataset. The authors have been able to resolve those that are straightforward to do [e.g. wrong tissue] but it doesn't mean that other problems have gone unnoticed. Given the relatively small sample size [compared to genetic studies for example] this is a high level of mix-ups that warrants a bit more discussion and checking.

*We reiterate that these issues stem from upstream sample handling/processing before reaching our lab. Roughly half of our mixups are sample swaps which will affect any study which uses the GTEx biobank, although we think that brain regions – which are impossible to distinguish after sectioning – might be more affected than other tissues. In other words, the sample mixups will affect any study using samples from the GTEx biobank, and the reviewer should extend their skepticism to any study using GTEx samples (although that’s not completely fair – we identified and fixed these issues). There is one issue which is study specific: the fact that some samples were shipped in overfull 96-well plates and material from one well could therefore overflow to other wells (See Table S3). This could be specific to the samples we received, or it could be reflective of an overall carelessness in the GTEx biobank. We have no idea, and we are not involved in running the biobank.*

*We have taken a path of complete transparency. We extensively checked our samples for genotype concordance and accurate PCA clustering by brain region and have provided this information and figures in the manuscript. We have been rigorous and forthright in describing our sample handling/processing/QC analyses. All we can do is work with what we were given and weed out the incorrect samples. We could have decided not to report these numbers and just omit the affected samples, a strategy which seems to have been employed by the main GTEx manuscript where we have not been able to find any details on sample mixups other than through personal communications with one or two groups which removed the questionable samples from further analyses.*

*The reviewer comment “*but it doesn't mean that other problems have gone unnoticed” *could be leveraged against* ***any*** *study. What we have done, is careful genotyping coupled with careful investigation of the (largely) tissue specific methylation marks. This is in contrast to many QC studies of large scale molecular profiling efforts which have revealed (for example) mismatched genotypes in* ***published*** *studies of, for example, 4% on average in worms (Zych et al 2017 PLOS ONE), 3% on average in human eQTL studies (with a single study having 23% mislabeled) (Westra et al 2011 Bioinformatics). In contrast, we would claim our study has 0% mismatch in the published data / samples.*

4. I still debate the unequivocal assumption that NeuN+ = neuronal. There is much evidence that this is not always the case. E.g. <https://www.ncbi.nlm.nih.gov/pubmed/17995928,> and I think the jury is still out about exactly how specific NeuN is. Perhaps simply say "neuron-enriched"? The authors words imply that sorting neun+ nuclei is a panacea. It is still a very broad population of cell-types and there will be considerable heterogeneity. It is highly plausible that specific neuronal cell populations are affected in schizophrenia - there is a huge literature on this. So it is not correct to assume that any between-individual heterogeneity in cell proportions is removed by doing this step - it's a very naïve assertion. In fact, some more refined cell-type estimates can be derived using some of the data from specific neuronal populations performed by several of the psychencode groups. I think this needs more work and more discussion.

*First, regarding our use of the word neuronal: All of the evidence, including the reference you cite, conclude that* in vitro *NeuN stains non-neuronal populations, but* in vivo *(aside from cerebellar Purkinje cells and dentate nucleus neurons, Cajal*–*Retzius cells in developing cerebellum, olfactory bulb mitral cells, retina photoreceptor cells, brain stem dorsal cochlear nucleus neurons, and sympathetic chain ganglion neurons along the spinal cord, none of which are present in our tissues) it only stains neurons. Additionally, we have shown in our previous publication that the largest source of variation is the NeuN+/NeuN- proportion, but of course this doesn’t account for other sources of variability. We acknowledge in the text that even withinthe NeuN+ population there are multiple subpopulations. We are not making case:control comparisons, which could rightly be particularly sensitive to neuronal subpopulation differences, but just comparing neuronal samples isolated from distinct brain regions from “normal” individuals. In no way is our data contradicting any study of SZ attributing effects to distinct neuronal populations; those studies are extremely interesting and of great value. To determine if there is residual variation in the proportion of NeuN- among our tissues, we implemented EpiDISH as recommended by the reviewer using 201 CpGs identified by Kozlenkov et al 2014 to distinguish NeuN+/NeuN- populations. To get to 201 CpGs, we first filtered for sites with |Db| > 0.7 resulting in 426 sites. We then eliminated any CpG that overlapped our 8-group and hippocampal DMRs. This step was performed to remove variation due to region-specific neuronal methylation differences to improve estimate accuracy. We find that all but 4 hippocampus samples are 100% neuronal. Three of the 4 have estimates > 97% neuronal and the last is estimated to be 87% neuronal. We have included these results in Fig. S2a (right) and have written the following in the main text.*

*Lines 259-272: As we have previously shown [12], much of the variability due to cell type heterogeneity is removed upon isolating NeuN+ nuclei from brain tissues. However, proportions of neuronal subpopulations between brain regions and among individuals could still contribute to variability measurements. Using EpiDISH [41], we estimated the NeuN+ proportions in our samples. For reference data we used sites of differential methylation between NeuN+ and NeuN- nuclei isolated from orbitofrontal cortex identified in Kozlenkov et al [42]. We filtered the 51,412 CpG sites they identified as*

*“neuronal undermethylated” and “glial undermethylated” for |Db| > 0.7 resulting in 426*

*sites. We then eliminated CpGs that overlapped DMRs identified in our 8-group and hippocampal analyses resulting in 201 reference CpGs. This step was critical to eliminate variation due to known region-specific neuronal methylation differences. We found that only four hippocampus samples had any evidence of glial (NeuN-) contamination thus providing independent validation of our sorting efficiency (****Fig. S2a****). Only one of these was less than 97% neuronal with an estimate of 87%.*

5. Re the efficiency of the FANS method, I still think this needs validation. Without getting too defensive, the authors could do this easily by applying a statistical deconvolution method such as CETS to their data. Hopefully the neuronal estimates from their data are 100%. Or they could look at DNAm in promoter regions of some key neuron-expressed genes.

*We have followed the reviewer’s suggestion. The results we present in Fig. S2a confirm our claims. See response to #4 above.*

6. I still strongly believe that the mQTL section should be removed. It really is very underpowered and prone to false positives. There is no way this can be construed as a robust analysis given the number of samples included in the genotype groups. The fact that the authors themselves note that they didn't mention it in the abstract highlights how they feel this is also exploratory.

*We have removed this analysis and instead elaborated on the comparisons with other VMR datasets from:*

1. *Garg et al (2018)*

[*https://journals.plos.org/plosgenetics/article?id=10.1371/journal.pgen.1007707#sec002*](https://journals.plos.org/plosgenetics/article?id=10.1371/journal.pgen.1007707#sec002)

2. *Gunasekara et al (2019)*

[*https://genomebiology.biomedcentral.com/articles/10.1186/s13059-019-1708-1*](https://genomebiology.biomedcentral.com/articles/10.1186/s13059-019-1708-1)

*We have also included an enrichment analysis of mQTLs identified in brain from multiple studies and found the highest enrichment in ubiquitous VMRs. This is shown as Figure 6b and described in the text as:*

*Lines 380-386: To examine possible genetic contributions to our observed methylation variability, we asked if mQTLs identified from brain tissues were enriched in our VMRs (****Fig. 6b****). The mQTL data we chose were generated using 450k arrays on samples from bulk hippocampus (n = 110) [55], fetal brain (n = 166) [6], and bulk temporal cortex (n = 44) that was also sorted into NeuN+ (n =18) and NeuN- (n = 22) nuclei [56]. We found the greatest enrichments in all datasets within our ubiquitous VMRs consistent with our assertion that methylation variability in this class of VMRs is genetically driven.*

7. "Our data suggests that most \TMRs arenot shared across all brain regions. This does not mean that the \TMRs are not genetically driven of course, but it means that methylation is only driven by genetics in some -but not all -brain regions". This is a very surprising result and remains so. The default has to be that there is some technical issue as major inter-individual variation generally results from genetic factors and is largely correlated across cells/tissues. It is known from repeated studies in the literature that genetically-driven DNA methylation variation is correlated across brain regions (and also between brain and periphery]. This section might benefit from an interpretation in the context of the work by Rob Waterland - e.g. see <https://genomebiology.biomedcentral.com/articles/10.1186/s13059-019-1708-1>

*We have looked at this particular reference in depth; however, the authors of this study first filtered their results on those bins that had high correlation across tissues, and call the resulting regions for CoRSIVs. The authors go on to state that “Previous studies have estimated that 15­20% of CpG sites in the human genome show tissue-specific interindividual variation (4,14). CoRSIVs are exceedingly rare by comparison, comprising only 0.1% of human genome.” The authors then subsequently establish that ~60% of CoRSIVs are genetically determined. However, it is true by definition that CoRSIVs are regions of variable methylation which are present in many tissues. This is in perfect agreement with our findings because these authors set out to specifically identify those regions of variation that were NOT tissue-specific. We did not do this and have instead catalogued both tissue-specific and shared interindividual variation.*

*We have also searched the literature for other work supporting the reviewer’s claim, and have been unable to identify it so far. Much of the literature focuses on regions of variable methylation which are present in multiple tissues (ie. the same as Waterland’s study) but this is really something quite different.*

8. "However, the kits available or measuring hydroxymethylation (including oxBS and TAB-seq) have never been successfully used in large sample studies." This is not true. There are several studies that have used the OxBS approach in large numbers of samples, including in brain - e.g. <https://www.ncbi.nlm.nih.gov/pmc/articles/PMC5604137/>

*The reviewer is correct and we were imprecise in saying they have not been performed with large sample sizes when we meant that this hasn’t been done at a genome-wide scale but predominately using arrays.*

9. "However, we are unable to provide a review link to this protected data." Why not? Without having access to this to check the analyses it is not [possible to fully review the paper. This goes against the submission standards expected in Genome Biology...

*We understand your frustration and share it, as we have been trying to get our data up on the GTEx portal and into dbGaP for many months through coordination with the GTEx LDACC. As part of a consortium, we have to follow the data deposition group’s lead on this.*

*However, given the delays – as well as the very reasonable insistence that data should be inspectable - we have now created a genome browser track of all our data for your use. It can be accessed by going to https.//*[*genome.ucsc.edu/cgi-bin/hgHubConnect#unlistedHubs*](http://genome.ucsc.edu/cgi-bin/hgHubConnect#unlistedHubs) *and loading https.//*[*egtex-wgbs.s3.amazonaws.com/hub.txt*](http://egtex-wgbs.s3.amazonaws.com/hub.txt.)*.*

*We have now included this link to our data in the manuscript.*

*Lines 791-793.” Data are made available as a UCSC track hub that can be accessed by*

*going to https.//*[*genome.ucsc.edu/cgi-bin/hgHubConnect#unlistedHubs*](http://genome.ucsc.edu/cgi-bin/hgHubConnect#unlistedHubs) *and loading https.//*[*egtex-wgbs.s3.amazonaws.com/hub.txt*](http://egtex-wgbs.s3.amazonaws.com/hub.txt)*. “*

**Third round of review**

Reviewer 2

The authors have done a good job with their revisions and I thank them for taking the comments made into consideration. I had a very minor comment on the abstract in that I don't think I agree with this statement "This significantly expands the knowledge of differential methylation across the brain by 10-fold" - it's quite subjective -- in terms of n's of people this is smaller by some way than other studies. Can this sentence be removed.

I have one totally optional suggestion as it relates to a paper I came across the other day looking at DNAm age in the human cortex - Brain. 2020 Dec 1;143(12):3763-3775. doi: 10.1093/brain/awaa334.

It would be very interesting to see whether DNAm age differs across brain regions, and the authors are in quite a unique position to do this. I realise the clock is designed for use on array data, but if the sites are coevered I think this would be interesting.

Thank you again for the efforts put into revising this paper.
